# Cognitive Intervention Targeting Autobiographical Memory Impairment in Patients With Schizophrenia Using a Wearable Camera: A Proof-of-Concept Study

**DOI:** 10.3389/fpsyt.2020.00397

**Published:** 2020-05-08

**Authors:** Romane Dassing, Mélissa C. Allé, Mathieu Cerbai, Alexandre Obrecht, Nicolas Meyer, Pierre Vidailhet, Jean-Marie Danion, Amaury C. Mengin, Fabrice Berna

**Affiliations:** ^1^ INSERM U1114 Neuropsychologie Cognitive et Physiopathologie de la Schizophrénie, Strasbourg, France; ^2^ Faculté de Médecine, Université de Strasbourg, Strasbourg, France; ^3^ Center on Autobiographical Memory Research, Department of Psychology and Behavioural Sciences, Aarhus University, Aarhus, Denmark; ^4^ Hôpitaux Universitaires de Strasbourg, Service de Psychiatrie, Santé Mentale et Addictologie, Strasbourg, France; ^5^ Hôpitaux Universitaires de Strasbourg, Département de Santé Publique, Strasbourg, France; ^6^ Université de Strasbourg, Laboratoire de Biostatistique et Informatique Médicale, iCUBE UMR 7357, Illkirch, France

**Keywords:** schizophrenia, autobiographical memory, cognitive remediation, wearable camera, rehabilitation

## Abstract

Autobiographical memory (AM) impairment in schizophrenia affects the richness of detail in personal memories and is one of the major predictors of patients’ social functioning. Despite the empirical evidence attributing these difficulties to a defective encoding process, cognitive remediation interventions targeting AM in schizophrenia often focus on the remote past, making it difficult to address the consequences of poor encoding. Our study evaluated the efficacy of an innovative approach using a wearable camera (NarrativeClip^®^) in reinforcing the encoding of recent daily life events in patients with schizophrenia. Seventeen patients with schizophrenia and 15 control participants wore the camera during four consecutive days. Then, memories of events experienced during these days were reinforced using different types of retrospective, i.e. interventions designed to promote a re-encoding of the event. We evaluated two types of retrospective using the camera pictures: a simple visual retrospective and a visual retrospective associated with a specific event-cueing (VisR+EC). These two techniques were compared to a verbal retrospective and to the absence of retrospective. Our results showed that the VisR+EC allowed patients to retrieve as many details as the control group at a two-week interval. However, patients’ memories remained impaired when a simple visual or a verbal retrospective was used. Our study provides encouraging results to foster the use of a wearable camera in individualized cognitive remediation programs for AM impairment in schizophrenia.

## Introduction

A large number of studies have documented the presence of cognitive impairments in patients with schizophrenia (1–3), and such impairments have come to be considered a core feature of this disorder. Although the entire set of cognitive functions seems to be affected in schizophrenia, these alterations are heterogeneous (3). Memory impairment is among the most pronounced cognitive deficits in schizophrenia (4) and goes beyond memory processes evaluated in laboratory, affecting complex cognitive functions such as autobiographical memory (5). Although the pure neurocognitive nature of these deficits is at some point discussed (6, 7), they have a concrete impact on patients’ daily functioning, often hindering patients’ access to a traditional educational or professional trajectory (8).

Autobiographical memory allows individuals to capture spatio-temporal, emotional, sensory, and cognitive representations of specific past personal events over long time intervals, which can extend up to several decades for particularly significant events (9–11). The retrieval of these episodic details is generally accompanied by a conscious recollection which confers the capacity to mentally travel back in time and re-experience a specific event. Autobiographical memory is a crucial function of human cognition, as it contributes to the emergence and construction of a coherent sense of identity (9, 12). It plays an important role in social relationships (13) and promotes a relevant anticipation of the future (14).

Several studies investigating autobiographical memory in schizophrenia have shown that patients have difficulty accessing unique past experiences in comparison to control subjects (15–19). Indeed, patients’ autobiographical memories are described as being “over-general”, more similar to summaries of repeated life experiences than to unique detailed events (20). Moreover, conscious recollection at the time of memory retrieval appears reduced in schizophrenia in comparison to controls (16, 21–23). A meta-analysis by Berna et al. (5) highlighted moderate to large effect sizes associated with these decreased specificity (*g* = -0.97), level of detail (*g* = -1.40), and conscious recollection (*g* = -0.62), all of which are key features of the episodicity level of autobiographical memory.

The functional mechanisms responsible for patients’ memory impairment have primarily been studied using standard laboratory episodic memory tasks. Although memory impairment was first attributed to a retrieval deficit (24, 25), empirical evidence has since emphasized the major implication of a defective encoding process in patients’ memory difficulties (26). Finally, autobiographical memory deficit in schizophrenia has been described as one of the major predictors of social functioning, surpassing the influence of both clinical symptoms and basic cognitive deficits (27). As such, it is one of the major therapeutic targets in the cognitive landscape of schizophrenia.

The antipsychotics used in schizophrenia to overcome clinical symptoms either have no particular effect or a deleterious effect on patients’ memory disorders (28). Psychological interventions have therefore begun to be developed in the past few decades, including cognitive intervention programs (29).

Reminiscence or “*life-review*” therapies (30) were among the first therapeutic programs aimed at stimulating autobiographical memory capabilities. Designed to promote the retrieval of vivid and detailed autobiographical past events, they usually target specific life periods, ranging from the past year to early childhood. Two studies (31, 32) have evaluated the efficacy of such programs, respectively the Autobiographical Reminiscence Therapy (REMau) (33) and the Life Review Therapy focusing on positive events (LRTspev) (34) in patients with schizophrenia. An improvement in the capacity to retrieve specific autobiographical memories was highlighted with both programs, and a greater number of details in patients’ recalled memories was observed with the LRTspev (32).

Another autobiographical memory intervention consists of applying a specific event-cueing at the time of memory retrieval (35). This specific event-cueing follows a series of questions inspired by the Memory Characteristics Questionnaire (36) and asks for different categories of episodic details (contextual, perceptual/sensory, temporal, cognitive, and emotional) not mentioned by participants during their free recall. Potheegadoo et al. (37) have shown its efficacy in patients with schizophrenia. Indeed, the use of specific event-cueing at retrieval allowed patients to reach a level of detail in memory recall similar to that of healthy subjects. Unfortunately, this was not true for the richness of perceptual/sensory, temporal, and contextual details, which remained reduced in patients despite the assistance of the event-cueing. The authors proposed that the event-cueing helped to overcome patients’ retrieval difficulties but did not alleviate the consequences of poor encoding of the event. It therefore seemed necessary to develop cognitive interventions to compensate this defective encoding process in schizophrenia.

Two studies have evaluated the effect of supporting memories of recent (less than a week old) daily life events on the encoding process in patients with schizophrenia using the diary method in a group setting (38, 39). This method asks patients to write down one or more recent events in a diary on a daily basis, using this tool as an external memory aid. Subsequently, patients are invited to share their memories with the other members of the group. The results of these two studies were conclusive, since they showed an improvement in memory specificity following the diary intervention that was maintained for up to three months (38). However, this method has several disadvantages: it is very time-consuming and it requires a high level of motivation, which can be difficult to mobilize in patients with schizophrenia (40). Furthermore, the material used (diary content) is limited by the accuracy of the patient’s memory, yet this is precisely the therapeutic target of the intervention, since the memories of patients with schizophrenia tend to be overly general. The number of cues patients provide may thus be insufficient to reflect their initial experiences. Finally, this diary method does not allow the experimenter to verify the accuracy of the content used as remediation material, and so altered or even false memories can be retrieved and trained during these interventions.

Considering the limitations of the diary method, several studies have begun to investigate memory using a wearable camera as a cognitive remediation tool for autobiographical memory disorders. The first device used for such purposes was the Sensecam^®^ camera, which can be worn around the neck and is typically programmed to take pictures automatically every 30 s. This tool does not require any particular intervention from the participant, which facilitates its use in patients with initiation or motivation difficulties. As using a wearable camera makes it possible to create dynamic slideshows with a very large number of pictures taken from a first-person perspective, the participant benefits from a cueing method very close to the initial lived experience (41, 42). Over the past decade, several studies have investigated the effect of viewing such event-related slideshows on autobiographical memory performance across multiple pathological situations (41–47). Results showed that the use of the Sensecam^®^ camera triggered the recollection of more specific and detailed memories, even in the case of severe amnesia. In addition, several studies have compared the use of a wearable camera to the diary method previously described (41, 43, 45, 46). Results showed that the improvement in autobiographical memory retrieval is more pronounced in the Sensecam^®^ condition. This inter-condition difference seems to expand over time, as shown by Woodberry et al. (46), who found memories to be up to three times more detailed in the Sensecam^®^ condition than in the diary condition at a three-month follow-up. Interestingly, Loveday and Conway (41) also highlighted an effect of the type of cueing method on the nature of the reported details, with an advantage for episodic details for events trained with the Sensecam^®^ pictures, and an advantage for general knowledge in the diary condition. In addition, several studies have suggested that viewing Sensecam^®^ pictures can trigger a “Proustian moment”, i.e. the intense retrieval of a specific memory including also non-sensory details (emotions, thoughts) experienced at the time of the initial experience, although not perceivable on the pictures (48). These previous studies have primarily assessed the effect of wearable cameras on autobiographical memory in neurological populations (e.g. post-encephalitic amnesia, traumatic brain injury, mild cognitive impairment, and Alzheimer’s disease).

Considering that deficient encoding processes may contribute to autobiographical memory impairment in patients with schizophrenia, we were interested in evaluating the relevance of material obtained with a wearable camera in fostering patients’ memories of recent daily life events. In order to collect the most ecological daily life events possible, we did not intervene at the time of the initial encoding of the events. For this reason, the interventions described in this study cannot be considered as direct interventions on encoding processes, but have rather been used to 1) reinforce the consolidation of the initially encoded experience by structuring its content and 2) promote a new and richer encoding of the lived event.

More precisely, the central aim of this proof-of-concept study was to test the efficacy of the NarrativeClip^®^ camera (a more recent version of the Sensecam^®^ camera) in improving the richness (i.e. number of details) and episodicity level with which patients with schizophrenia recall recent personal experiences. We compared the effect of two interventions using the NarrativeClip^®^ camera: 1) a simple visual retrospective involving only the viewing of a slideshow of pictures collected during the day and 2) a visual retrospective associating this viewing with the specific event-cueing used by Potheegadoo et al. (37). These two methods were then compared to a verbal retrospective without viewing the pictures of the day and to the absence of retrospective. In light of the literature, we expected that the visual retrospectives using the NarrativeClip^®^ pictures would promote a greater improvement in the number of details recalled in comparison to the verbal retrospective and, even more so, to the absence of retrospective. Moreover, we predicted that patients with schizophrenia would benefit more from the visual retrospective with event-cueing than from the simple visual retrospective, considering the dual modality (visual and verbal) of this condition and the active involvement required of the participant.

## Materials and Methods

### Participants

Thirty-two participants took part in the study. These included a group of 17 outpatients (six women) diagnosed with schizophrenia according to the DSM-5 criteria (49), who were recruited from the Strasbourg University Hospital Psychiatric Department, and a comparison group of 15 control participants (seven women). Patients and controls did not differ in either age or level of schooling. All the patients had been clinically stabilized under antipsychotic treatment for at least two months. We did not include patients treated with benzodiazepines due to their adverse effects on memory (50). None of the study’s participants had a history of neurological or major somatic illness, a current alcohol or substance abuse problem, or a current severe depressive episode, as defined by a score higher than 6 on the Calgary Depression Scale for Schizophrenia (CDSS) (51) for patients and by a score higher than 9 on the Beck Depression Inventory (BDI) (52) for controls. Furthermore, control participants had no history of psychiatric disorder. In addition, we measured pre-morbid IQ in each group using a validated French version of the National Adult Reading Test (f-NART) (53). Current IQ was measured using a validated abbreviated version of the Wechsler Adult Intelligence Scale – third edition (WAIS-III) (54), which included three subtests: vocabulary, matrix reasoning, and arithmetic (55). Participants with a current IQ score below 70 were excluded from the study.

This study was approved by the Committee for the Protection of Persons of Paris X (reference 2013-A00402-43), and all participants gave their written informed consent in accordance with the Declaration of Helsinki.

### Materials and Procedure

**Table 1** presents the clinical and cognitive measures administered to participants.

**Table 1:** Means (M) and standard deviations (SD) of sociodemographic, clinical, and cognitive measures for patients with schizophrenia and controls.

		Control participants	Patients with schizophrenia	Statistics
		n = 15	n = 17	
		*M (SD)*	*M (SD)*	*Pr* (P > C)
**Sociodemographic characteristics**				
Gender (number of women, %)		7 (46.7)	6 (35.3)	0.262
Age (years)		40.6 (10.58)	40.24 (10.29)	0.739
Level of schooling (years)		13.67 (2.02)	12.29 (2.62)	0.063
**Clinical variables**				
PANSS^a^	Total Scale	–	57.63 (19.3)	
	Positive Scale	–	12.94 (5.63)	
	Negative Scale	–	18 (9.44)	
Depression	BDI^b^	3.07 (2.31)	–	
	CDSS^c^	–	2.38 (1.61)	
Duration of illness (years)		–	13.29 (8.78)	
Chlorpromazine equivalents (mg)		–	322.25 (191.61)	
**General intelligence**				
		*Total IQ* ^d^ *scores*		
f-NART^e^ (premorbid IQ^d^)		106.87 (7.04)	106.73 (8.75)	0.938
Short version of WAIS-III (current IQ^d^)		96.48 (11.87)	90.05 (11.27)	0.793
**Cognitive assessments**				
		*Standard scores*		
WMS-III^f^	Family Scenes I	9.67 (2.72)	6.65 (4.06)	**0.013**
	Family Scenes II	9.87 (2.59)	6.71 (3.79)	**0.007**
	Family Scenes (percentage of retention)	10.00 (2.88)	8.88 (3.64)	0.192
	Spatial Memory	9.57 (1.99)	8.75 (3.15)	0.221
		*Z-scores*		
TMT^g^	B - A (time)	-0.48 (0.97)	-0.70 (1.34)	0.305
	B - A (number of errors)	-0.64 (1.80)	-0.85 (2.36)	0.397
Fluency	Phonological	-0.41 (0.52)	-0.16 (0.69)	0.849
	Semantic	-0.27 (0.76)	-0.89 (0.90)	**0.031**

Results are presented as the probability that patients’ scores to be higher than the controls’ scores is above 0: Pr(P > C). A large Pr(P > C) value (e.g., > .95, > .975, or > .99) must be interpreted as indicating higher values for patients compared with controls. A small value of Pr(P > C) (e.g., < .05, < .025, or < .01) reflects lower values for patients compared with controls. It is worth noting that the probability Pr(P > C) can be interpreted as 1 – Pr(P < C). Probability values near 1, and near 0, both indicate a meaningful effect of the group.

^a^Positive And Negative Syndrome Scale for Schizophrenia. ^b^Beck Depression Inventory. ^c^Calgary Depression Scale for Schizophrenia. ^d^Intelligence Quotient. ^e^French National Adult Reading Test. ^f^Wechsler Memory Scale – third edition. ^g^Trail Making Test.

#### Clinical Assessments

To assess the severity of clinical symptoms of schizophrenia, we used the Positive and Negative Syndrome Scale (PANSS) (56) in patients.

#### Cognitive Assessments

To investigate long-term memory performance, we used the Family Scenes subtest of the Wechsler Memory Scale-III (WMS-III) (57). We selected this standardized task because its material is more similar to what our study evaluated (recall of autobiographical events) than the lists of words usually employed in conventional verbal memory tests. In addition, knowing that long-term memory encoding and retrieval processes depend on executive processes, we measured working memory performance using the WMS-III’s Spatial Memory subtest. Participants’ mental flexibility was also measured using a verbal fluency task (58), which assessed both the phonological and semantic aspects of fluency, and the non-verbal Trail Making Test (TMT) (59).

#### Experimental Protocol

During the first visit, we asked each participant to wear the camera (NarrativeClip^®^) during a short trip with the experimenter. The purpose of this was firstly to familiarize the participant with the tool and estimate any possible associated discomfort or anxiety, and secondly to train the participant to position the device correctly on his/her clothing.

Subsequently, if the person agreed to participate in the study, he/she was invited to carry the camera for four consecutive days, for a minimum of seven hours a day. The events experienced during each day were collected and allocated to a different type of retrospective (i.e. intervention). Then, two weeks later, a memory test was conducted to evaluate the robustness of these events’ memories and thus the respective effect of each type of retrospective.

##### Events Collection and Interventions

**Figure 1:**
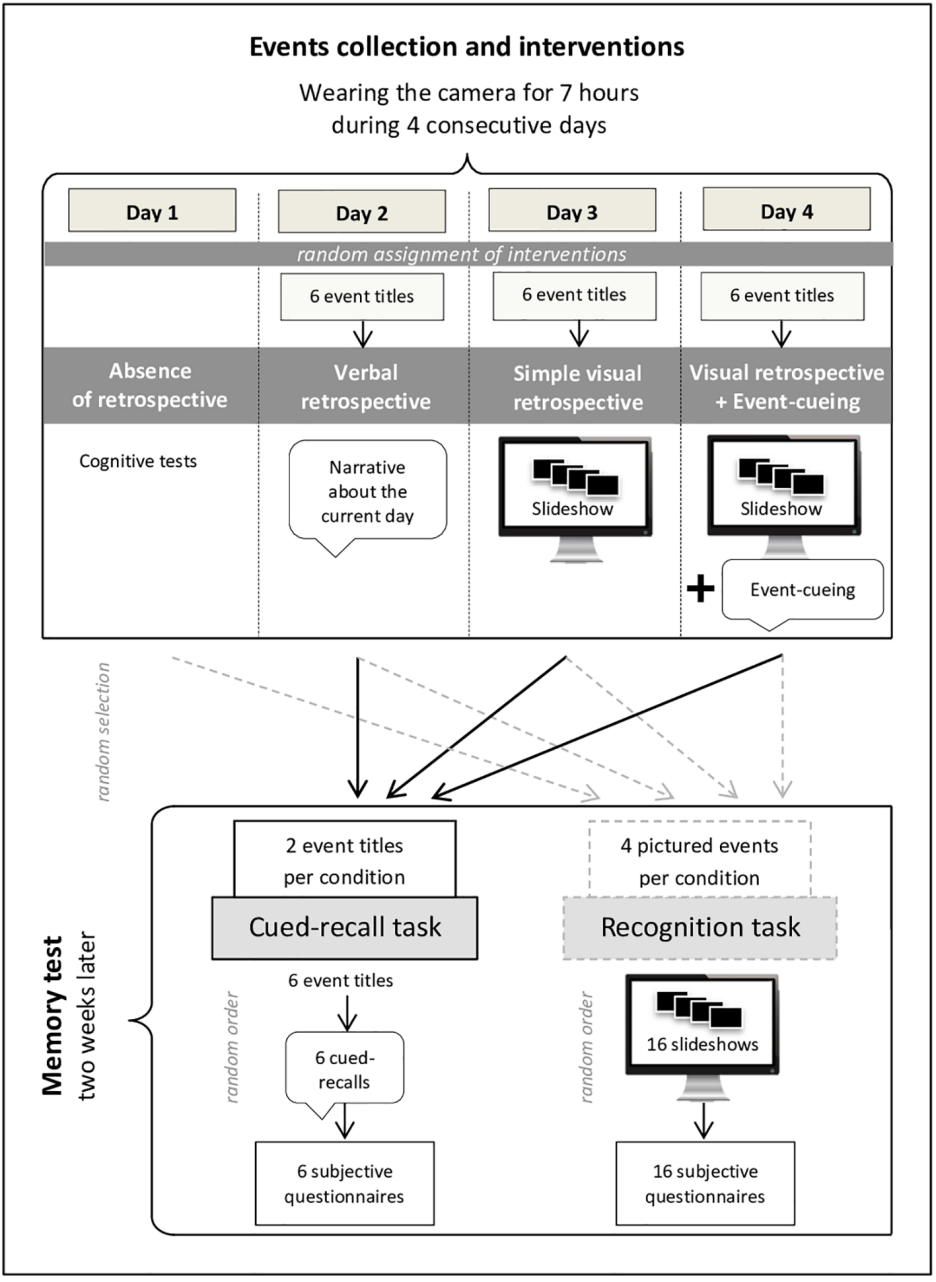
Schematic of the experimental protocol’s design, including the events collection phase and the memory test.

At the end of each day, we collected the day’s pictures and each time the memories of the day were subjected to a different type of intervention (the order of which being randomly assigned; for a schematic of the events collection and interventions, see **Figure 1**).

Here is one of the possible combinations of interventions:

###### Day 1: Absence of retrospective – control condition (AbsR)

The participant is invited to carry out the vocabulary and matrix reasoning subtests of the WAIS-III (54), without making any reference to the course of his/her day.

###### Day 2: Verbal retrospective (VerR)

The experimenter asks the participant to give a title to six of the most significant events experienced during the day. The participant is invited to orally recount the events experienced during the day in chronological order, making sure that his/her story includes the six pre-selected events. As described above, the production of a written content requires a high level of motivation. In order to avoid disadvantaging the group of patients, and since this narrative will not be used as a cue in the memory test, we therefore opted for an oral task.

###### Day 3: Simple visual retrospective (VisR)

Similarly to the verbal condition, the participant is asked to give a title to six of the most significant events experienced during the day. Subsequently, a slideshow comprising the pictures collected during his/her day is presented to the participant, without any verbal intervention from the experimenter. The participant is invited to silently pay attention to the pictures of his/her day. The pictures scroll at a rate of one picture per second.

###### Day 4: Visual retrospective associated with event-cueing (VisR+EC)

The participant is asked to give a title to six of the most significant events experienced during the day. Subsequently, a slideshow comprising the pictures collected during the day is presented to him/her. The participant is asked to stop the slideshow at the time of each of the six pre-selected events. With the picture of the event displayed before him/her, the participant is then questioned in depth about the event, following the procedure of Levine et al. (35). The questions follow a predefined order and aim to encourage the participant to recall visual and non-visual details of the scene (see the detailed procedure in **Supplementary Material S1**).

The participants of both groups were able to select six events experienced during their day, without any major difficulty for the three conditions with retrospective (VerR, VisR, and VisR+EC). The 6*3 event titles collected in these conditions were then used for the memory test two weeks later. Two out of the six event titles were randomly selected in each condition to be used as cues in the cued-recall task. The remaining event titles were used to create the slideshows of the recognition task (for a schematic of the event selection, see **Figure 1**).

At the end of these four days of collecting events, a questionnaire was presented to each participant, which evaluated his/her subjective impressions of the NarrativeClip^®^ tool. This questionnaire consisted of two central questions, measuring 1) the level of constraint associated with the camera and 2) the usability of the camera, using Likert scales ranging from 1 (constraining/difficult to use) to 7 (pleasant/easy to use).

##### Memory Test

The memory test was conducted two weeks after the last day of wearing the camera and included two different tasks (for a schematic of the memory test, see **Figure 1**):

###### Cued-Recall Task

The aim of the cued-recall task was to measure the level of detail remembered by the participants two weeks after the initial events.

For this task, 2*3 events titles were randomly selected from the 6*3 events identified by the participant during the verbal, simple visual, and visual retrospectives associated with event-cueing (two events per condition). Since the condition with no retrospective was not subjected to event selection by the participant, it was not included in this task. The experimenter gave the written title of each of these events as a cue to the participant, who was directly invited to recall aloud all the information he/she remembered concerning the event. All these autobiographical memories were recorded and then transcribed.

All the verbatim transcripts were analyzed according to the scoring method of Levine et al. (35). This objective method translates qualitative content into quantitative content, adding and classifying the various details present in the narratives. Indeed, each piece of information provided by the participant was counted and classified in a specific category, starting with a distinction between internal and external details, that is, details related or unrelated to the selected event (see descriptions and examples presented in **Table 2**).

**Table 2:** Examples of details for each of the categories described in the scoring method of the Autobiographical Interview (Levine et al., 2002).

Event Recollection								
Internal details					External details			
directly related to the selected specific event					not related to the selected specific event			
Event details	Place details	Time details	Perceptual details	Emotion/Thought details	Other event details	Semantic details	Repetitions	Other details
Actions, people and weather	From a country to a part of a room	From a period of life to a precise schedule	Related to the five senses or perceptions of a position/duration	Mental state at the time of the event	Belonging to another specific event	General knowledge about the world and oneself	Second occurrence of a detail	Comments or metacognitive reflections
*I went to withdraw money.*	*It was in a mall.*	*It was around* *10:30 a.m.*	*The cash dispenser screen was blue.*	*I thought of the balance of my account.*	*The last time I came to this mall, I bought a sweater.*	*This shopping center contains fifty or so stores.*	—	*I do not remember that well…*

All narratives were analyzed by the first author (RD), and a trained second rater (fourth author, AO) analyzed a random selection of 60% of the transcripts. At the time of the analysis of the memories, the raters were naive to the group to which the participants belonged and the experimental condition. An intra-class correlation coefficient was calculated to assess the consistency of the two contributors’ ratings. This analysis showed excellent inter-rater reliability, with Cronbach’s alpha of 0.97 and 0.91 for internal and external detail ratings, respectively.

After each event recall, the participant was asked to complete a subjective questionnaire to evaluate various characteristics related to his/her memory on a seven-point Likert-type scale, among which were its episodicity level, its associated valence and emotional intensity, and its importance for the participant (see the detailed questionnaire in **Supplementary Material S2**).

###### Recognition Task

In the recognition phase, the purpose was to investigate in which extent participants subjectively estimated the robustness of their memories when we gave them the visual content of each experience.

Sixteen events were randomly selected from the verbal, simple visual, and visual retrospective associated with event-cueing conditions, as well as from the control condition (four events per condition). For the conditions with retrospective, the 4*3 selected events were those that had not been discussed during the cued recall. For the control condition (without retrospective), the experimenter himself selected four events from the picture bank of the day.

These 16 events were mixed with eight “lure events” created by the experimenter using the wearable camera and therefore not personally experienced by the participant. These events allowed us to verify that each participant was able to recognize whether he/she had experienced an event personally or not, both in the control group and the group of patients with schizophrenia.

Each of these events was presented in random order to the participant in a short slideshow including approximately 20 pictures. If the participant felt that he/she had not lived the event presented, he/she was not given a questionnaire, and we proceeded to the following event. If the participant recognized the event as personally experienced, he/she was invited to complete the same subjective questionnaire as in the cued-recall task, evaluating several characteristics related to his/her memory.

### Statistical Analyses

Bayesian methods were used to analyze the study’s data (60). Between-group comparisons of sociodemographic (age, level of education), IQ and cognitive measures were performed using univariate linear regressions. The number of details recalled in the cued-recall task was analyzed using a mixed Poisson regression model with group (patients vs. controls) and type of retrospective (verbal vs. simple visual vs. visual associated with event-cueing) as predictors. Because the ratings of the subjective memory characteristics presented in both the cued recall and the recognition tasks were bounded variables and not normally distributed, mixed Beta regression models were calculated using the same predictors (group and type of retrospective) to analyze these data.

In all analyses, we computed the mean difference of each parameter tested and its 95% credible interval (indicated as CI95%). We then calculated the probability that the score of each measure was higher in the patient group than in the control group [indicated as *Pr*(P > C)]. Correlation coefficients are provided with their means and 95% posterior credible intervals and the probability that this coefficient is larger than 0 [indicated as *Pr*(*r* > 0)]. Probabilities higher than 95% or lower than 5% were considered meaningful.

We used informative priors to perform each of these regressions; priors were estimated according to previous studies that 1) demonstrated autobiographical memory impairments in schizophrenia (5, 15, 16, 21, 61) and 2) have recently successfully used a wearable camera for autobiographical memory remediation in amnesic, brain-damaged, or mildly cognitively impaired patients (41, 43–46). Subsequently, to evaluate the robustness of our findings concerning the transcript ratings, non-informative priors and pessimistic priors (where the values of the informative priors were entered in the regression analyses, but in the opposite direction to what was expected) were used (see details in **Supplementary Material S3**).

A burn-in of 5,000 iterations followed by 100,000 iterations was used for each of the three chains, yielding a final 300,000-iteration sample for retrieving the characteristics of the posterior distribution. Convergence of the MCMC sample chains was checked graphically and was observed in each case. All computations were done in the R computing environment with the required additional packages (in particular r2jags).

## Results

### Clinical and Cognitive Measures

Concerning sociodemographic characteristics, our two groups did not differ in terms of age, gender, or level of education (see **Table 1**). Similarly, the two groups did not differ in their premorbid and current IQ levels. With regard to cognitive measures, the patient group presented poorer performances than the control participants in information storage (both short- and long-term) and executive functioning, in the context of semantic fluency. Correlation analyses showed no relevant association between cognitive or clinical evaluations and measures of autobiographical memory detail in either group.

### Experimental Protocol

No participants expressed dissatisfaction regarding the NarrativeClip^®^ camera during the study. Indeed, they estimated it to be relatively simple (*M* = 6.28, *SD* = 1.28) and pleasant (*M* = 5.08, *SD* = 1.51) (range: 1–7) to use. Scores did not differ between the groups (all *Pr*s(patients > controls) between 0.14 and 0.23).

#### Measures of Autobiographical Memory Detail

In total, 141 autobiographical event transcripts were included in the analysis, of which 63 were from the patient group (22 for the verbal retrospective condition, 21 for the simple visual retrospective condition, and 20 for the visual retrospective with event-cueing condition) and 78 from the control group (26 for each type of retrospective).

##### Internal Details

**Figure 2:**
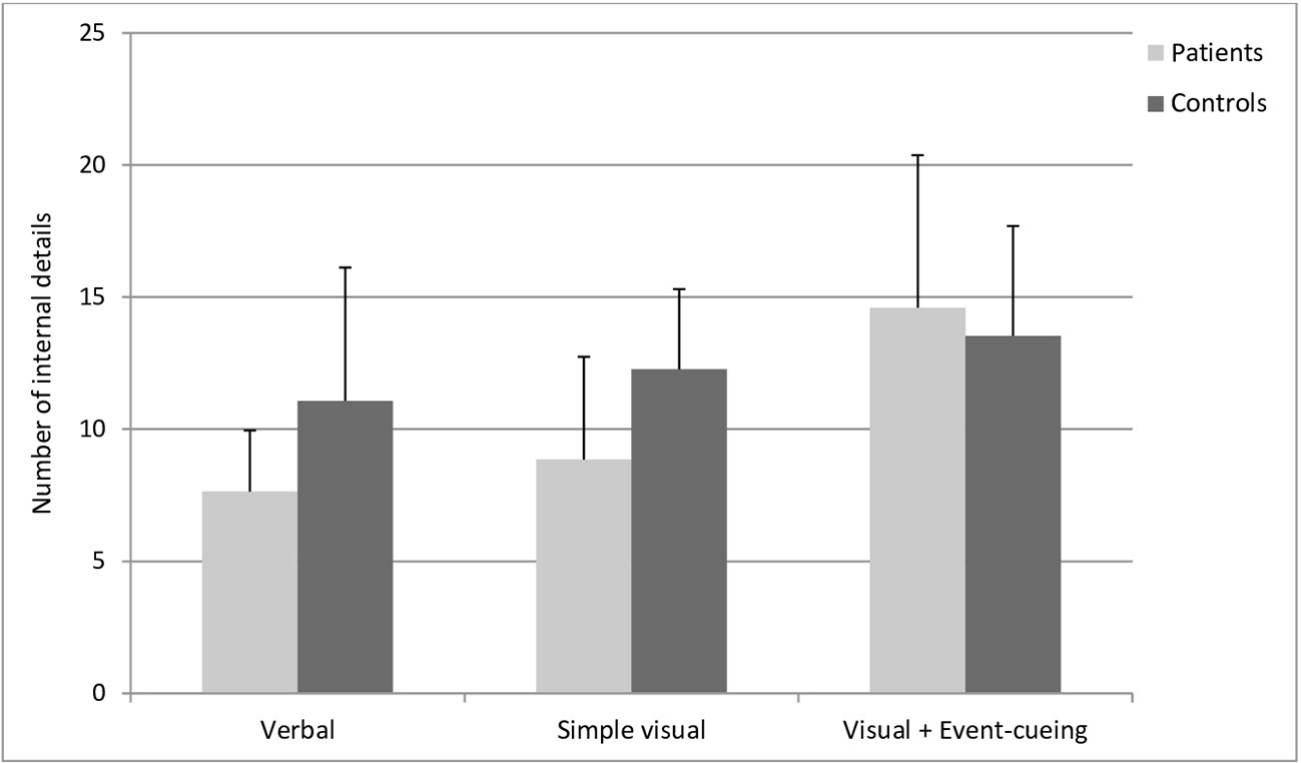
Mean numbers of internal details in participants’ recalls in the cued recall task, analyzed according to the scoring method of the Autobiographical Interview (35). A larger number of internal details was found in the Visual + Event cueing condition, compared to the Verbal condition. A meaningful group by type of retrospective interaction was found, with an overall smaller number of internal details in the group of patients compared to controls, except for the Visual + Event-cueing condition.

A smaller number of internal details was observed in the patients’ memories compared to those of the controls [OR=0.67, CI95%: 0.43–1.03, *Pr*(patients > controls) = 0.03], and a larger number of internal details was found in the VisR+EC condition than in the VerR condition [OR=1.21, CI95%: 1.03–1.41, *Pr*(VisR+EC > VerR) > 0.99]. Other pairwise comparisons between conditions showed that the number of internal details was higher in VisR+EC vs. VisR vs. VerR, but the probabilities that these differences differed from 0 were only between 0.87 and 0.88. Finally, a meaningful group by type of retrospective interaction was found [OR=1.56, CI95%: 1.22–2.00, *Pr*(OR > 1) > 0.99], showing that the number of internal details was lower in the patient group than in the control group in the VerR condition, but not in the VisR+EC condition (see **Figure 2**).

##### External Details

**Figure 3:**
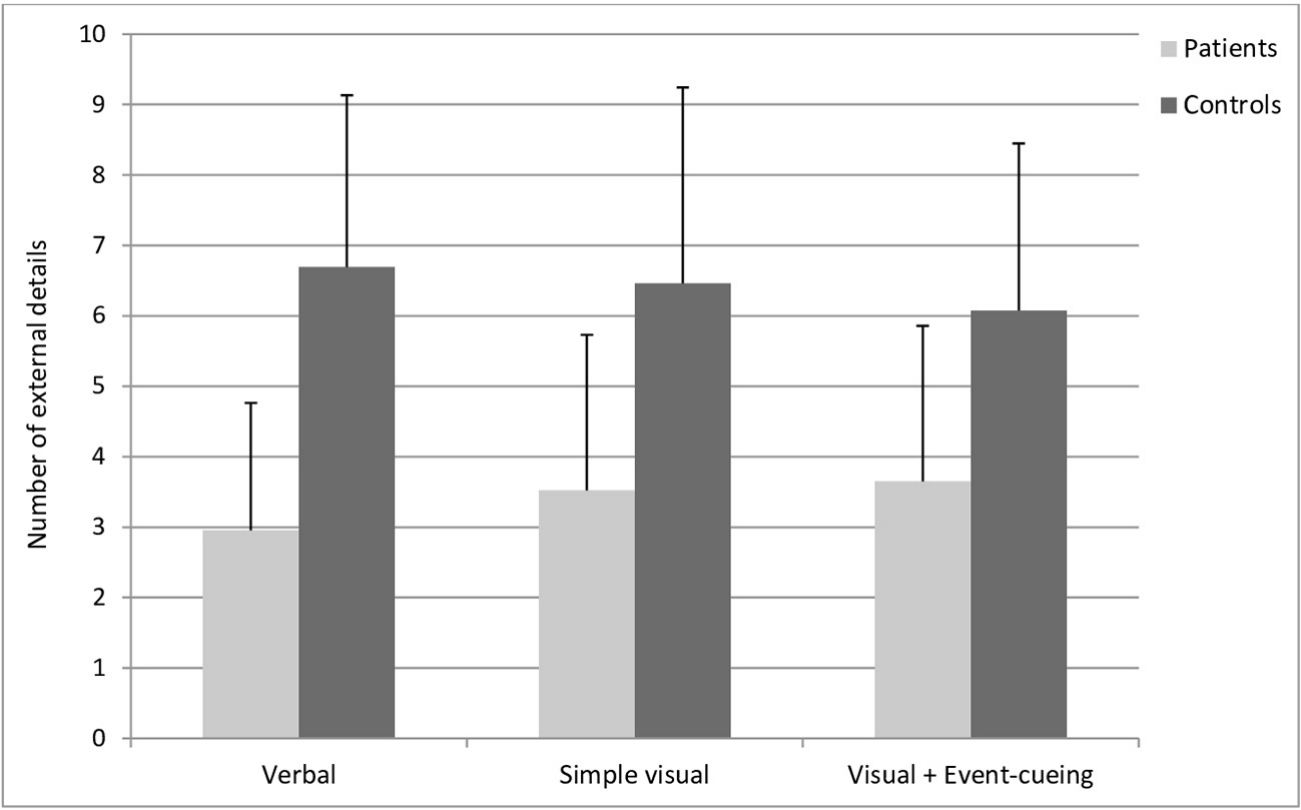
Mean numbers of external details in participants’ recalls in the cued recall task, analyzed according to the scoring method of the Autobiographical Interview (35). A meaningful group effect was observed showing a smaller level of external details in the patient group, compared to the control group. No relevant differences were observed across the different types of retrospective.

A smaller number of external details was found in the patients’ memories compared to those of the controls [OR=0.6, CI95%: 0.18–0.71, *Pr*(patients > controls) = 0.002]. No meaningful differences in score were observed across the different types of retrospective [all *Pr*s(OR > 1) between 0.20 and 0.38]. The interaction between group and type of retrospective was also not meaningful [all *Pr*s(OR > 1) were between 0.82 and 0.89] (see **Figure 3**).

#### Ratings of Subjective Autobiographical Memory Characteristics

##### Cued-Recall Task

**Figure 4:**
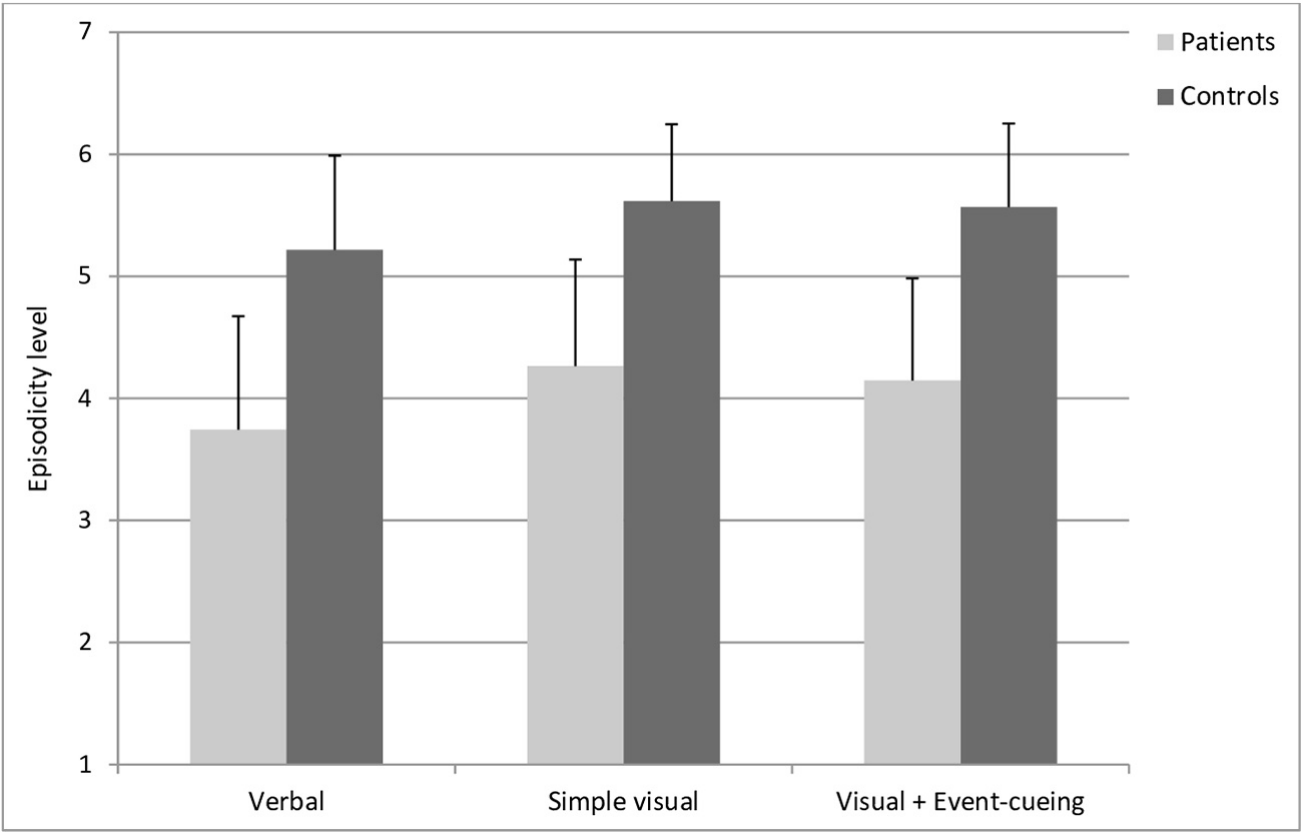
Mean episodicity level (and standard deviations) evaluated by the participants during the cued recall task. These results highlighted lower scores of episodicity in the patient group compared to controls, but no relevant differences across the different types of retrospective.

We computed a composite episodicity score by averaging the scores for two ratings: the ability to relive the event mentally and the level of detail (see **Figure 4**). The results indicated lower scores in the patient group compared to the control group [OR=0.36, CI95%: 0.18-0.69, *Pr*(patients > controls) = 0.001], but scores did not differ across the various types of retrospective [all *Pr*s(OR > 1) between 0.41 and 0.82]. Similarly, there was no meaningful interaction between group and type of retrospective [all *Pr*s(OR > 1) between 0.62 and 0.69].

Subsequently, each remaining item was analyzed separately (see **Table 3**). Patients displayed a reduced ability to relive the event mentally [OR=0.30, CI95%: 0.16–0.58, *Pr*(patients > controls) < 0.001] and lower levels of memory detail [OR=0.46, CI95%: 0.23–0.91, *Pr*(patients > controls) = 0.01] and emotional intensity [OR=0.49, CI95%: 0.23–0.99, *Pr*(patients > controls) = 0.02] compared to controls. The results also showed that the memory detail scores were higher in the VisR condition than the VerR condition [OR=1.58, CI95%: 0.92–2.73, *Pr*(VisR > VerR) = 0.95]. The scores in the other conditions did not differ [all *Pr*s(OR > 1) between 0.40 and 0.70]. Finally, no relevant interaction between group and type of retrospective was observed [all *Pr*s(OR > 1) between 0.09 and 0.87].

**Table 3:** Means (M) and standard deviations (SD) of memories characteristics subjectively evaluated by participants during the cued recall task.

	Control participants n=15			Patients with schizophrenia n=17		
	Verbal	Simple visual	Visual + Event-cueing	Verbal	Simple visual	Visual + Event-cueing
	*n=30*	*n=30*	*n=30*	*n=34*	*n=34*	*n=34*
	*M (SD)*	*M (SD)*	*M (SD)*	*M (SD)*	*M (SD)*	*M (SD)*
**Level of details**	4.93 (1.95)	5.60 (1.33)	5.50 (1.46)	3.79 (1.78)	4.15 (1.81)	4.18 (1.77)
**Ability to mentally relive**	5.50 (1.25)	5.63 (1.27)	5.63 (1.38)	3.70 (2.07)	4.38 (1.79)	4.12 (1.79)
**Emotional valence**	1.43 (1.28)	1.43 (1.30)	1.67 (1.15)	0.79 (1.41)	0.79 (1.47)	0.29 (1.71)
**Emotional intensity**	4.27 (1.41)	4.43 (1.38)	4.53 (1.68)	3.30 (1.19)	3.68 (1.61)	3.53 (1.46)
**Importance**	4.17 (1.64)	4.57 (1.59)	4.30 (1.84)	3.33 (1.53)	3.76 (1.79)	3.79 (1.75)

Each of these characteristics was assessed by the participants on Likert scales ranging from 1 to 7, except emotional valence which was estimated on a Likert scale ranging from -3 to 3.

##### Recognition Task

Only one lure event was recognized as having been personally experienced, and 11 of the 768 events assessed were classified as non-personal events (eight in the patient group, three in the control group). This result confirms the ability of all participants to discriminate personally experienced events from lure events.

**Figure 5:**
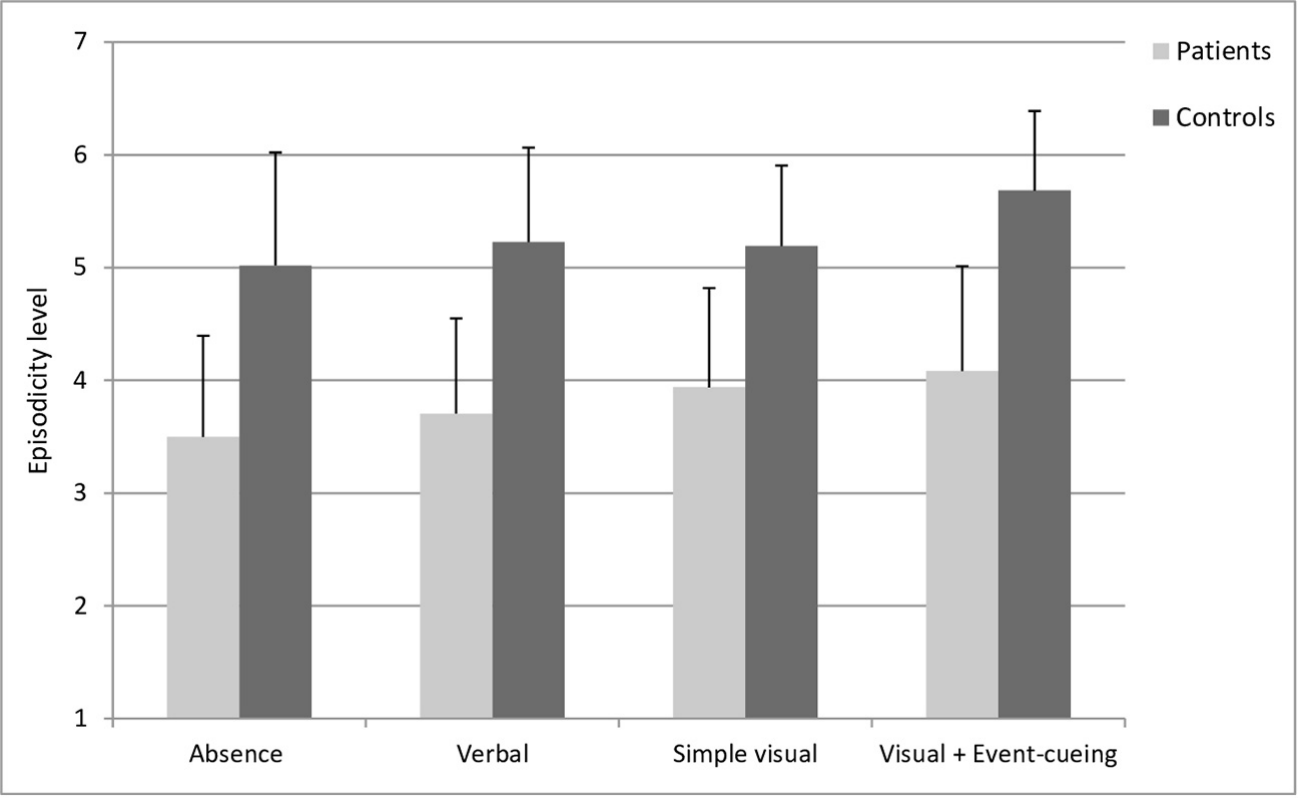
Mean episodicity level (and standard deviations) evaluated by the participants during the recognition task. Episodicity levels were estimated as lower in the patient group compared to the control group. No other relevant differences were observed across the conditions.

Lower episodicity scores were observed in the patient compared to the control group [OR=0.36, CI95%: 0.2–0.66, *Pr*(patients > controls) < 0.001] (see **Figure 5**). Episodicity scores were higher in the visual retrospective with event-cueing condition than in the absence of retrospective condition [OR=1.66, CI95%: 1.14–2.42, *Pr*(VisR+EC > AbsR) > 0.99]. The scores in the other conditions did not differ [all *Pr*s(OR > 1) between 0.51 and 0.94], and no group by condition interaction was observed [all *Pr*s(OR > 1) between 0.46 and 0.76].

Regarding the remaining single items (see **Table 4**), patients displayed lower scores in the ability to relive the event mentally [OR=0.39, CI95%: 0.21–0.70, *Pr*(patients > controls) = 0.001] and in the level of detail [OR=0.35, CI95%: 0.20–0.64, *Pr*(patients > controls) = 0.001] than control participants. Similarly, ratings of emotional valence were lower in patients than in controls [OR = 0.51, CI95%: 0.27–0.95, *Pr*(patients > controls) = 0.02], showing patients’ tendency to judge event memories more negatively than controls did. The pairwise comparisons between the experimental conditions demonstrated higher estimations of the ability to relive the event mentally [OR=1.66, CI95%: 1.12–2.43, *Pr*(VisR+EC > AbsR) = 0.99] and of the level of detail [OR=1.65, CI95%: 1.12–2.45, *Pr*(VisR+EC > AbsR) = 0.99] in the visual with event-cueing condition compared to the absence of retrospective condition. Scores in the ability to mentally relive the event were also higher in the VisR+EC condition compared to the VerR condition [OR=1.37, CI95%: 0.93–2.07, *Pr*(VisR+EC > VerR) = 0.95]. No other relevant differences were observed across the remaining types of retrospective [all *Pr*s(OR > 1) between 0.08 and 0.93]. Finally, there was no interaction between group and type of retrospective [all *Pr*s(OR > 1) between 0.17 and 0.94].

**Table 4:** Means (M) and standard deviations (SD) of memories characteristics subjectively evaluated by participants during the recognition task.

	Control participants				Patients with schizophrenia			
	n=15				n=17			
	Absence	Verbal	Simple visual	Visual + Event-cueing	Absence	Verbal	Simple visual	Visual + Event-cueing
	*n=60*	*n=60*	*n=60*	*n=60*	*n=68*	*n=68*	*n=68*	*n=68*
	*M (SD)*	*M (SD)*	*M (SD)*	*M (SD)*	*M (SD)*	*M (SD)*	*M (SD)*	*M (SD)*
**Level of details**	5.00 (2.00)	5.29 (1.72)	5.18 (1.49)	5.67 (1.40)	3.27 (1.81)	3.59 (1.76)	3.91 (1.80)	4.10 (1.93)
**Ability to mentally relive**	5.04 (2.05)	5.16 (1.71)	5.20 (1.49)	5.70 (1.44)	3.58 (1.87)	3.82 (1.82)	3.97 (1.80)	4.06 (1.86)
**Emotional valence**	1.09 (1.40)	1.16 (1.41)	0.88 (1.46)	1.17 (1.32)	0.24 (1.45)	0.34 (1.53)	0.51 (1.48)	0.33 (1.50)
**Emotional intensity**	4.28 (1.47)	4.15 (1.22)	4.20 (1.70)	4.10 (1.68)	3.63 (1.52)	3.30 (1.41)	3.66 (1.70)	3.63 (1.54)
**Importance**	3.67 (1.88)	4.00 (1.63)	3.72 (1.75)	3.73 (1.87)	3.68 (1.89)	3.61 (1.73)	3.57 (1.79)	3.67 (1.66)

Each of these characteristics was assessed by the participants on Likert scales ranging from 1 to 7, except emotional valence which was estimated on a Likert scale ranging from -3 to 3.

### Sensitivity Analyses

To test the robustness of the statistical analyses, we performed sensitivity analyses using non-informative and pessimistic priors concerning the two types of detail (internal and external) in the objective evaluations. The estimated coefficients remained unchanged, suggesting that the coefficient estimations were primarily driven by the data collected and not by our expected results.

## Discussion

The aim of the present proof-of-concept study was to explore whether and how an external memory aid, such as pictures taken by a wearable camera, can enhance the richness of patients with schizophrenia’s memories of recent personally experienced events. Our results showed that strengthening these memories with a visual retrospective method combined with event-cueing allowed patients to retrieve as many internal details as the control group at a two-week interval. This improvement was however not found when patients were asked to subjectively assess the vividness of their memories. Furthermore, the level of detail of patients’ memories remained impaired when a simple visual retrospective or a verbal retrospective was used. To our knowledge, this is the first study to investigate the efficacy of a wearable camera as a tool for the remediation of memories of recent personal events in patients with schizophrenia.

### Measures of Autobiographical Memory Detail

First of all, reduced richness of internal and external detail was observed in the patients’ compared to the controls’ memories in the simple verbal or visual retrospectives conditions. This observation corroborates existing findings on autobiographical memory in schizophrenia (15–19), which report difficulties for patients in accessing episodic details related to a past personal event. A meta-analysis by Berna et al. (5) has also highlighted a large effect size of -1.40 associated with this lower level of detail in patients’ memories. Our results extend existing knowledge by showing that these deficits also affect the memory of recent personal events, as studies on autobiographical memory in schizophrenia have typically investigated remote personal events that happened months or years before.

What is however surprising and new in the present study is the lack of difference between the verbal and the simple visual retrospectives in terms of internal details recalled. Our initial hypothesis predicted a larger number of details to be recalled for memories trained with the simple visual method, based on the literature on wearable cameras used in a cognitive remediation context. Indeed, the effectiveness of this type of tool in comparison to verbal interventions in improving retrieval of past personal experiences has previously been demonstrated (41, 43, 45, 46). Nevertheless, our study suggests that the simple use of pictures as re-encoding material without specific event-cueing does not improve the level of detail more than a verbal retrospective at a two-week interval.

Firstly, this result could be explained by the use of different methodological parameters in our study compared to previous studies. In the existing literature, the majority of studies (43–46) have operationalized memory enhancement using a set of approximately 15 details pre-established by a third person (typically the patient’s spouse). Indeed, patients’ recalls were represented as a percentage relative to these pre-defined details, which could reach 100% if the participant recalled all the details initially chosen by the third person. In our study, the evaluation of the retrospectives’ effect was based directly on the number of details recalled by the patient, according to the rating method of Levine et al. (35). Loveday and Conway (41) used a similar rating method by counting the number of details in the patient’s memories. However, in their study, the cued recall was carried out directly following the viewing of the slideshow (which constituted the cueing), while our participants were only given the title of the event during the cued-recall task. Indeed, we only showed the slideshow during the event collection phase, as soon as possible after the event encoding (two weeks before the cued-recall task), and only for the events related to these types of retrospective. A first explanatory hypothesis would be that in the previous studies, the benefit of the simple viewing of the pictures without any additional cueing was dependent on the rating method used (percentage of recall related to a pre-established list of details in previous studies vs. number of recalled details in ours) and on the interval between the slideshow viewing and the participant’s recall (immediate recall vs. two week delay).

A complementary explanation of our results can be provided by work done in the field of retrieval practice. In fact, one may consider that our verbal and simple visual retrospectives did not require the same degree of involvement from the participant. While in the simple visual retrospective task the participant was only asked to passively watch the slideshow (and thus to restudy the material to be learned), the verbal retrospective task required additional engagement insofar as the participant tested his/her own memory by organizing the events of his/her day in as complete a narrative as possible. Roediger et al. (62) demonstrated that the retention of material is strengthened when the individual is invited to test his/her learning compared to when the material is “simply” re-studied. This testing effect has been established in the healthy population (63) as well as in several clinical populations (64–69), including schizophrenia (70). Indeed, patients, similarly to controls, showed better recall performances when the material to be remembered (a list of word pairs) was subjected to a test in comparison to a restudying condition. Although to our knowledge the literature on the testing effect has not yet been evaluated in autobiographical memory tasks, it seems appropriate to question its potential implications for our results. Indeed, the low cognitive effort involved in the passive viewing of the slideshow certainly did not make the simple visual retrospective effective enough to distinguish itself from the verbal retrospective, which can be viewed as an active memory test of the events experienced during the day. In addition, previous literature has shown that a retention interval of more than one day favours the occurrence of a testing effect (63). These findings are consistent with our results, since our protocol employed a long retention interval (two weeks). To bypass this possible bias, it would have been necessary to include a “passive” verbal retrospective. However, this is unfortunately impossible to conceive without the involvement of a third person, who would be required to narrate to the participant the events he/she had experienced during the day.

It is worth stressing that memory performance did not differ across the patient and control groups when the memories were trained with the visual retrospective coupled to event-cueing, suggesting that patients may have normalized their memory performances. This result aligns with that of Potheegadoo et al. (37) which also showed a normalization of patients’ performance following a specific cueing procedure. However, one major distinction from Potheegadoo’s study (37) is that the event-cueing procedure was used during a “re-encoding” phase and not at the time of event retrieval. Thus, our result tentatively suggests that our intervention made it possible to compensate the deficient encoding of real-world events in patients when those events were retrieved a few days later. It however remains difficult to explain the weak effect of the simple visual retrospective, in view of its efficacy when coupled with the event-cueing. In order to more finely discriminate the contribution of the verbal and visual retrospective and better grasp the cueing procedure’s contribution to memory improvement, further studies would benefit from adding a similar event-cueing procedure to the verbal retrospective condition.

### Ratings of Subjective Autobiographical Memory Characteristics

Lower overall memory episodicity was found in the patient group compared to the control group for both the cued-recall and recognition tasks. This result aligns with patients’ difficulty in accessing rich details related to daily life events and in mentally reliving these events (5, 23). Regarding inter-condition differences, episodicity was evaluated as higher for the events trained with the visual retrospective associated with event-cueing compared to the control condition (absence of retrospective) in the recognition task. No other differences were found between the different types of retrospective, neither in the cued-recall task nor in the recognition task. We could therefore interpret this higher episodicity rating as the result of the patients having developed familiarity with the slideshows, which were presented again in the recognition task. In other words, the participants may have remembered the corresponding slideshow that they had seen previously in the events collection phase two weeks earlier rather than accurately re-experiencing the original event. In line with this, Loveday and Conway (41) reported that although the post-cue self-rating of memory vivacity was higher than the initial rating, no difference was found between the different cueing methods (diary vs. wearable camera). Loveday and Conway’s study (41) and the present work, taken together, indicate the difficulty of highlighting an inter-condition difference in subjective ratings, even if the participant has just watched the slideshow. This observation raises the question of the sensitivity to change of subjective measures of autobiographical memory characteristics.

### Limitations

Several limitations of our work should be acknowledged. Keeping in mind that our study was conceived as a proof-of-concept, it is premature to conclude the utility of wearable cameras in improving autobiographical memory of daily life events in patients with schizophrenia. In fact, a limited number of events was considered here over a brief period of time, and these events mostly consisted of trivial daily life events with limited self-relevance. Furthermore, we did not investigate the presence or absence of a memory complaint in our patients. One may assume that patients with memory complaints would benefit to a greater extent from these memory interventions than patients without such complaints. In addition, since we did not target patients in demand for cognitive intervention, we did not investigate to which extent they were likely to use a camera to improve their memory capacities. These points should be addressed in future studies.

### Conclusion and Future Perspectives

This first proof-of-concept study in schizophrenia has substantial clinical implications, since it provides encouraging results concerning both the usefulness and acceptability of wearable cameras for improving autobiographical memory in these patients. The next step is to investigate whether patients with schizophrenia reporting autobiographical memory difficulties are likely to use this type of camera in a therapeutic setting. In line with previous studies conducted in a variety of clinical conditions (41–47), future studies should envision personalized cognitive remediation programs reinforcing autobiographical memories over longer periods of time. Inspired by previous experimental protocols (43, 46), it would also be crucial to train selected events repeatedly with a verbal retrospective based on the diary method (in which the re-encoding material would be a written summary of the event produced by the patient himself/herself) and other selected events with a visual retrospective based on the pictures taken by the wearable camera and comparing these two approaches to a non-interventional control condition. Furthermore, to deepen the evaluation of the respective effects of both verbal and visual retrospectives, it would be wise to associate both with the event-cueing used in the current study. To ensure the therapeutic value of these cognitive remediation programs, it will be necessary to carry out measures of generalization and transfer of the interventions to untrained cognitive and functional areas. All in all, since remembering personally experienced events allows us to clarify our sense of identity (71) and helps us to mentally anticipate future events (14), one can reasonably expect positive outcomes of these interventions, such as a reduction in disability and an increased quality of life in patients. However, these points need to be further investigated in future.

## Data Availability Statement

The datasets generated for this study are available on request to the corresponding author.

## Ethics Statement

The studies involving human participants were reviewed and approved by Committee for the Protection of Persons of Paris X (reference 2013-A00402-43). The patients/participants provided their written informed consent to participate in this study.

## Author Contributions

MA, J-MD, and FB designed the study. RD, MA, and MC collected the data. RD, AO, and FB conducted the analyses. NM supervised the method and statistical analyses. RD wrote the first draft of the manuscript. MA, PV, J-MD, AM, and FB provided substantial contributions to the manuscript. All authors read and approved the final version of the manuscript. RD and MA contributed equally to the study.

## Funding

This work was supported by INSERM (Institut National de la Santé et de la Recherche Médicale), France and by the University Hospital of Strasbourg under a Grant for Young researcher (API 2012 – HUS N°5539).

## Conflict of Interest

The authors declare that the research was conducted in the absence of any commercial or financial relationships that could be construed as a potential conflict of interest.
