# Supplementary material for: Cognitive Intervention Targeting Autobiographical Memory Impairment in Patients With Schizophrenia Using a Wearable Camera: A Proof-of-Concept Study
Source: Front Psychiatry. 2020 May 8;11:397. doi: 10.3389/fpsyt.2020.00397 (PMC7247825; doi:10.3389/fpsyt.2020.00397)
Supplement: Supplementary file 1 [file DataSheet_1.pdf]

COGNITIVE INTERVENTION TARGETING AUTOBIOGRAPHICAL MEMORY IMPAIRMENT IN PATIENTS WITH SCHIZOPHRENIA USING A WEARABLE CAMERA: A PROOF-OF-CONCEPT STUDY.

Supplementary Materials

S1. Questions of the event-cueing procedure, inspired by the specific probe proposed by Levine et al. (2002).

**Contextual details**<br>At what time of day did the event occur?<br>Can you describe the place you were in?<br>Was the general framework familiar to you?<br>Was this type of event usual for you?<br>Who was present during the event?
**Sensory-perceptual details**<br>*These questions were only asked when they were considered relevant in the context of the event.*<br>Can you describe some visual details of this event?<br>Can you describe some auditory details of this event?<br>Can you describe some olfactory details of this event?<br>Can you describe some gustatory details of this event?<br>Can you describe the spatial arrangement of the people around you?
**Emotion/Thought details**<br>Do you remember how you felt at the time of the event?<br>Can you describe the intensity of your feelings at the time of the event?<br>Did you think about anything in particular during the event?<br>Do you remember the conversations that took place during the event?

S2. Variables, items and response scales of the subjective questionnaire.

**Level of details**	My memory of the event is…	*From 1 = Very vague to 7 = Very detailed*
**Ability to mentally relive**	I am able to mentally relive this event.	*From 1 = Not at all to 7 = Perfectly*
**Emotional valence**	When I remember this event, my feelings are...	*From -3 = Very negative to 3 = Very positive*
**Emotional intensity**	When I remember this event, my feelings are...	*From 1 = Not very intense to 7 = Very intense*
**Importance**	To me, this event is…	*From 1 = Trivial to 7 = Very important*

S3. Estimation of the informative priors.

Estimation of the informative priors for the condition effect

Estimations were mostly based on the following papers

- Woodberry et al. 2015 showing 62% of memories recalled with wearable camera after one viewing vs. 50% with diary
- Browne et al. 2011: 66% of memories recalled with wearable camera after 2 weeks vs. 38% with diary
- And we used **64% and 44%** as mean percentage of memories recalled with wearable camera and diary respectively

Informative priors (i.e. estimated entered in the Beta regression) were calculated using the formulas below and are reported in the right part of the table. We estimated that effects across to be of similar strength in the all paired comparisons (i.e. AbsR vs. VerR, VerR vs. VisR, VisR vs. VisR+EC, VerR vs. VisR+EC etc…)

- M_e_alpha was calculated using log((M_p_diary /(1-M_p_diary))
- SD_e_alpha was calculated using (CI_e_alpha 97.5% - CI_e_alpha 2.5%) / (2*1.96)
- Precision_e_alpha was calculated using 1/SD_e_alpha^2
- M_e_theta was calculated using log((M_p_diary + M_p_wearcam)/(1-M_p_ diary - M_p_ wearcam))
- SD_e_theta was calculated using (CI_e_theta 97.5% - CI_et_heta 2.5%) / (2*1.96)
- Precision_e_theta was calculated using 1/SD_e_theta^2

		**Data From previous studies**					**estimated parameters for the Beta regression**				
		M_p	*SD_p*	*CI_p*		*parameters*	*M_e*	*CI_e*		*SD_e*	*Precision_e*
				2.5%	97.5%			2.5%	97.5%		
condition	diary	0.44	0.085	0.273	0.607	**alpha**	**-0.241**	-0.977	0.433	0.344	**8.451**
	wearcam	0.64	0.028	0.585	0.695		0.575	-0.344	0.823	0.126	62.63
	difference	0.20	0.113	-0.021	0.421	**theta**	**-1.386**	-3.892	-0.317	0.546	**3.358**

Note. M_p = Mean of the known parameter; SD_p = standard deviation of the known parameter; CI_p = Credible Interval of the known parameter; M_e = Mean of the estimated parameter; SD_e = standard deviation of the estimated parameter; CI_p = Credible Interval of the estimated parameter; Precision_e = precision of the estimated parameter (precision = 1/SD^2)

These priors were then entered in the Beta regression as follows (jags model in R used in our analyses):

```
model<-function(){
  for (i in 1:N){
    details[i]~dbeta(a[i], b[i])
    a[i]<- mu[i]*gamma
    b[i]<- (1-mu[i])*gamma
    logit (mu[i]) <- alpha + theta[condition[i]] + priorsuj[suj[i]]
  }
  for(n in 1:NSuj) {
    priorsuj[n]~ dnorm(0,tau) }
  tau~dgamma(0.1,0.1)

  alpha~dnorm(M_e_alpha,Precision_e_alpha)
  theta[1]<-0
  theta[2]~dnorm(M_e_theta,Precision_e_theta)
  OR<-exp(theta[2])
  PrOR<-step(OR-1)
  gamma~dgamma(0.01,0.01)
}
```

Estimation of the informative priors for the group effect

The means and SD for groups were based on the meta-analysis by Berna et al. (2015) that reported hedge's g for memory specificity (g = -0.97), level of details (g = -1.40) and conscious recollection (g = -0.62). In our case, our priors were estimated using g between 0.8 and 1.

Given that:

- mean_patients = mean_control – diff_group
- and that: Hedge's g = (mean_patients - mean_control) / sqr[(sd_patients + sd_controls)/2]
- That is: Hedge's g = (diff_group) / √[(sd_patients + sd_controls)/2]
- Then: diff_group = Hedge's g * √ [(sd_patients + sd_controls)/2]

Therefore, the coef_group used in the following equations was based on estimations of diff_group

For the variable "details" for instance, sd_patients and sd_controls were comprised between 1.2 and 2.0, so that (based on hedgde's g of 0.8 and 1), coef_group was comprised between 0.91 and 1.41.
